# Butenolide Insecticide Flupyradifurone Affects Honey Bee Worker Antiviral Immunity and Survival

**DOI:** 10.3389/finsc.2022.907555

**Published:** 2022-07-11

**Authors:** Gyan P. Harwood, Vincent Prayugo, Adam G. Dolezal

**Affiliations:** Department of Entomology, University of Illinois at Urbana-Champaign, Urbana, IL, United States

**Keywords:** honey bees, virus, pesticides, flupyradifurone, clothianidin, toxicity, seasonality

## Abstract

Honey bees face many environmental stressors, including exposure to pesticides and pathogens. A novel butenolide pesticide, flupyradifurone, was recently introduced to the US and shown to have a bee-friendly toxicity profile. Like the much-scrutinized neonicotinoids that preceded it, flupyradifurone targets the insect nervous system. Some neonicotinoids have been shown to interfere with antiviral immunity, which raised the concern that similar effects may be observed with flupyradifurone. In this study, we investigated how flupyradifurone and a neonicotinoid, clothianidin, affect the ability of honey bee workers to combat an infection of *Israeli acute paralysis virus* (IAPV). We exposed workers to field-realistic doses of the pesticides either with or without co-exposure with the virus, and then tracked survival and changes in viral titers. We repeated the experiment in the spring and fall to look for any seasonal effects. We found that flupyradifurone caused elevated mortality in the fall, but it did not lead to increased virus-induced mortality. Flupyradifurone also appeared to affect virus clearance, as bees co-exposed to the pesticide and virus tended to have higher viral titers after 48 hours than those exposed to the virus alone. Clothianidin had no effect on viral titers, and it actually appeared to increase resistance to viral infection in spring bees.

## Introduction

Honey bees are key agricultural pollinators, adding billions of dollars in increased crop yields (1), yet American beekeepers regularly lose over half their colonies annually (2). There are myriad biotic and abiotic stressors that contribute to these high annual losses, including exposure to pesticides, transmission of viruses and other pathogens, and poor nutrition (3–7). What is more, multiple stressors can occur simultaneously and have additive or synergistic effects on honey bee health and productivity (4, 8). In agricultural landscapes, honey bees are exposed to many classes of pesticides, some of which can exert direct effects on mortality, and others which can elicit sublethal effects that impair important physiological pathways (reviewed in (9)). Perhaps the best-studied example is the neonicotinoids. Since their introduction 30 years ago, neonicotinoid insecticides have received greater scrutiny for their sublethal effects on pollinators than other classes of insecticides, likely due to their rapid and widespread adoption by growers, and their ability to be taken up by plants systemically. Neonicotinoids can persist in plant tissues and the surrounding environment (10, 11), exposing off-target organisms to chronic sublethal doses. In an effort to protect pollinators, several neonicotinoids and other pesticides have been banned or restricted in some regions or countries (12–14).

There has been a push in recent years to develop new chemistries that are safer to honey bees and other pollinators. One such pesticide is flupyradifurone (the active ingredient in Sivanto^®^), a novel type of butenolide insecticide publicized as being safe for bees (15). Flupyradifurone is a systemic insecticide that, like neonicotinoids, targets nicotinic acetylcholine receptors (nAChRs) in the nervous system (16). Neonicotinoid insecticides have been shown to produce many sublethal effects on honey bees, including impairment of immune response. In addition to other pathogens, honey bees are beset by dozens of viruses, which can be vectored by parasitic *Varroa destructor* mites or transmitted horizontally and vertically between nestmates (17, 18). The majority of surveyed honey bee colonies harbor viral infections at subclinical levels (19, 20), but these infections can become clinical if stressors impede the bees’ abilities to suppress viral replication (21, 22). Honey bees use several innate immune system pathways to fight viral infection, but pesticides can disrupt these pathways (4). For example, studies show that exposure to the neonicotinoid clothianidin can suppress expression of a key transcription factor used in antiviral immunity and lead to higher replication of *Deformed wing virus* (DWV) in honey bees (23). Neonicotinoid exposure can also reduce the phagocytotic function of hemocytes (insect immune cells) (24, 25), thereby compromising an insect’s ability to eliminate virus-infected cells (26). It is important to note that all of these effects were only observed in studies occurring after these insecticides were registered and in use in many countries.

Studies from regulatory bodies and industry scientists show that flupyradifurone has low acute toxicity to honey bees, with minimal impact on mortality and productivity when used under recommended guidelines (27–29). Given flupyradifurone’s recent introduction to the US market, however, there are limited data available on more subtle effects to honey bee biology, and concerns have been raised regarding its safety for bees. High doses of flupyradifurone can increase oxidative stress (30) and affect honey bee taste, cognition, and motor skills (31, 32), while field-realistic doses can impair behavior and affect survival and immunity (33–35). Flupyradifurone exposure has been shown to increase expression of several honey bee immune genes (36), and lead to higher pathogen loads of the microsporidian *Nosema ceranae* in some colonies (37). Thus, as with neonicotinoids, it is imperative to consider not only acute lethal effects of flupyradifurone but also sublethal effects that may be more difficult to track. One clear current gap is our understanding of how flupyradifurone affects response to virus infection.

Proper evaluation of pesticide safety also requires consideration of the season in which the trials take place (38–40). Outside of winter, individual honey bee workers usually live for less than two months, but the colony as a whole is actively foraging from early spring to fall (or varying periods, depending on local environments). Over the course of the year, needs of the colony shift, from high population growth and colony proliferation in the spring, to the cessation of foraging and preparation for overwintering in the fall. This is accompanied by corresponding changes in worker physiology (41), meaning bees sampled in the spring differ from those sampled in the fall. Moreover, there is a seasonal shift in flowering species available for forage (42, 43). The nectar and pollen consumed by workers and fed to developing larvae contain plant phytochemicals that can stimulate detoxification pathways, which can affect how tolerant bees are to pesticides. For example, phytochemical consumption can increase bees’ resistance to some pesticides but not others (44, 45). Moreover, some phytochemicals can stimulate immune pathways and make bees more resistant to viral infection (46, 47). Flupyradifurone has already been shown to have seasonally-dependent toxicity to honey bees, where bees in southern California are more sensitive to flupyradifurone in the summer than in the early spring (33).

Here, we examined how exposure to flupyradifurone or the neonicotinoid clothianidin affected response to virus infection in honey bee workers. We hypothesized that, like in other studies of neonicotinoids and DWV (23), sublethal exposure to dietary insecticides reduces the ability of honey bees to respond to a virus infection. Further, we also hypothesized that responses to our treatments would differ across seasons. To test these hypotheses, we used *Israeli acute paralysis virus* (IAPV) as a model virus (48). IAPV can cause a debilitating disease that can be lethal to all honey bee castes and development stages (49–52), and has been implicated in colony failure (53). Further, it can be used for repeatable assays on survivorship that have been shown to be affected by different dietary treatments (7, 47, 54). Thus, we used IAPV treatments to assess the interaction of virus infection with flupyradifurone and clothianidin, and measured treatment-dependent mortality in both the spring and the fall to look for any seasonal effects. In addition to tracking survivorship, we also measured IAPV titers to determine whether either of the pesticides affects the rate at which the virus replicates or is cleared by the host.

### Methods

#### Bees

Bee colonies were maintained at the University of Illinois Bee Research Facility in Champaign County, Illinois, using standard beekeeping practices. Newly emerged (<24 hrs old) adult worker bees were acquired from 5 colonies by removing brood frames and placing them in an incubator overnight at 33°C and 60% relative humidity as described in previous work (7, 47). The emergent workers were then mixed and placed in clear plastic cages (10 x 10 x 8 cm) containing 35 individuals each and subjected to 1 of 6 treatment diets (see below). Cages of bees were placed back in an incubator with the same conditions as before and remained there for 7 days. A 7-day trial duration was chosen because virus-induced mortality peaks 24 – 48 hr after inoculation before survival levels off after, and we are focused on how pesticides affect virus-induced mortality during this time span. Previous studies have also found that IAPV-induced mortality or persistent IAPV infection can be observed well in 7 or fewer days (7, 47, 48, 50, 54). Throughout the experiments, bee mortality was monitored daily and dead bees were removed. The experiment was repeated twice, once in the fall (October) and once in late spring (June). In the fall trial we used n=10 cages per treatment group, while in the spring we increased this to n=15. The 5 donor colonies used for acquiring newly emerged workers differed between fall and spring trials, but in both instances all donor hives were healthy at the time of sampling and were led by queens from the same genetic source stock. They contained low Varroa mite populations (0-3 mites per 300 bees in regular alcohol wash sampling), had good nutritional resources, and had no overt signs of pathogen infection.

### Pesticides

Both clothianidin and flupyradifurone were purchased from SigmaAldrich (PESTANAL^®^ analytical standard, #33589 and #37050, respectively). They were dissolved in a solvent (acetonitrile, Alfa Aesar #42311) and stored in -20°C until later use. When incorporated into the 30% sucrose feed, they were diluted to field-relevant dosages of 10 ppb for clothianidin and 4 ppm for flupyradifurone. These dosages have been used elsewhere and are the approximate concentrations of each pesticide in the nectar of seed-treated canola fields, or else the concentration observed in nectar carried by foragers in their honey stomachs in such fields (23, 29, 34, 35, 55).

### IAPV

The IAPV inoculum was produced as described previously (47, 48), and prepared identically as in Hsieh et al., 2020. Briefly, white-eyed pupae were injected with a 1% solution of IAPV, and the virus was allowed to replicate for several days. The pupae were then harvested, and the viral particles were extracted and purified using standard protocols. As in (47), the inoculum contained an estimated 9.86 X 10^7^ IAPV genome equivalents in 100ng purified RNA (quantified *via* RT-qPCR) and was composed of 99.79% IAPV particles, with trace amounts of DWV, *Black queen cell virus* (BQCV), and *Sacbrood virus* (SBV). The inoculum was stored in -80°C until the trials, at which point it was diluted in 30% sucrose to a sublethal dose.

### Treatment Diets

Caged bees were subjected to 1 of 6 treatment diets: sucrose only (control), clothianidin, flupyradifurone, virus (IAPV), clothianidin+virus, and flupyradifurone+virus. The sucrose control and virus-only treatment were spiked with solvent at an equivalent concentration as the other treatment diets. The treatment diets were administered in 2 stages. In the first stage, cages receiving a pesticide treatment were given a small dish containing the pesticide dissolved in 600 mL of 30% sucrose (w/v), while all remaining cages received the same volume of 30% sucrose alone. Bees were allowed to feed for 2 hours, during which time they consumed all the food. In the second stage, virus-treated cages received a sublethal dose of IAPV in 600 mL of 30% sucrose, while all remaining cages received the 30% sucrose alone. The sublethal virus dose (0.5% of the virus stock inoculum) was determined in preliminary trials and typically resulted in 30-60% mortality by 48 hrs post inoculation (48). After viral inoculation, all cages were allowed to feed *ad libitum* from a drip feeder of 30% sucrose. For pesticide-treated cages, their diets were spiked with their assigned pesticide in the same concentration as stage 1 of the inoculation, meaning these treatment groups consumed pesticides for 7 days.

### Viral Titers

During the spring trial, we collected 1 bee per cage per day for the first 3 days of the trial (N = 162 total; n = 39-45 per treatment group), with the second collection day corresponding to the highest observed virus-induced mortality (48 hrs post inoculation). Due to work restrictions caused by the COVID-19 pandemic, we were unable to collect live samples of bees from the fall trials. All bees were alive at the time of sampling in the spring trial and we haphazardly collected workers which displayed no overt disease symptoms. Sampled bees were snap frozen in liquid nitrogen and stored in -80°C for future processing. RNA from each individual bee was extracted with a Qiagen RNeasy kit (#74106) and DNase-treated with a Qiagen RNase-free DNase kit (#79256) following standard protocols. Treated RNA samples were then loaded onto a 384 well plate using a one-step RT-qPCR kit (Applied Biosystems #4389986) and analyzed using a QuantStudio 6 real-time PCR instrument (Applied Biosystems). Samples were amplified over 40 cycles using the following parameters: 95°C for 15 sec, 60°C for 60 sec. A standard curve was used to estimate the quantity of virus particles. This approach has a theoretical limit of detection of approximately 200 virus particles per reaction (54), and used IAPV primers described therein.

### Statistics

For survival data, we used pairwise Cox proportional hazard mixed-effects models using cage ID as a random factor and Benjamini-Hochberg corrections for multiple comparisons. Such proportional hazard models only compare between 2 groups, thus necessitating the use of pairwise comparisons rather than a more holistic model with all treatments included simultaneously.

For pairwise comparisons, we first compared all virus-treated groups to all non-virus-treated groups to confirm that viral inoculation reduced survival. Next, we examined non-virus treatments and compared between each pesticide treatment and the sucrose control to look for pesticide-induced mortality. Finally, we examined virus treatments and compared each pesticide+virus treatment to the virus-only control to determine whether co-exposure to a pesticide alters pesticide-induced mortality.

For virus quantification, we first log_10_ transformed virus quantities obtained from the RT-qPCR. The data were binomially distributed, with highly infected individuals having >10^6^ viral copies per 200 ng of RNA and most remaining individuals having <10^4^ copies. We thus opted to use pairwise binomial exact tests with Benjamini-Hochberg corrections for multiple comparisons, in which we classified individuals as having either high-level or low-level infections. We chose a threshold of 10^5^ viral copies for distinguishing between high-level and low-level infections. This threshold was determined based on the natural separation in the data and previous findings by our group (56), in which colonies that were healthy (i.e., minimal Varroa mite infestation, no overt signs of disease, high brood production, etc.) could still have background IAPV infection, and covertly infected individuals therein had a maximum of approximately 10^5^ viral copies per 200ng of RNA. While bees from those colonies were sampled and analyzed as a large group, and there is a possibility some samples could have contained a heterogenous mixture of uninfected and highly infected individuals, it is notable that the natural cutoff in our experimental data roughly matches the field sample measurements.

We elected not to perform pairwise comparisons between the 2 pesticides due to the large (1000-fold) difference in their concentrations. All analyses were performed in R version 4.0.5 using the base package, “surv”, “survminer”, and “ggplot2”.

## Results

### Survivorship

**Figure 1:**
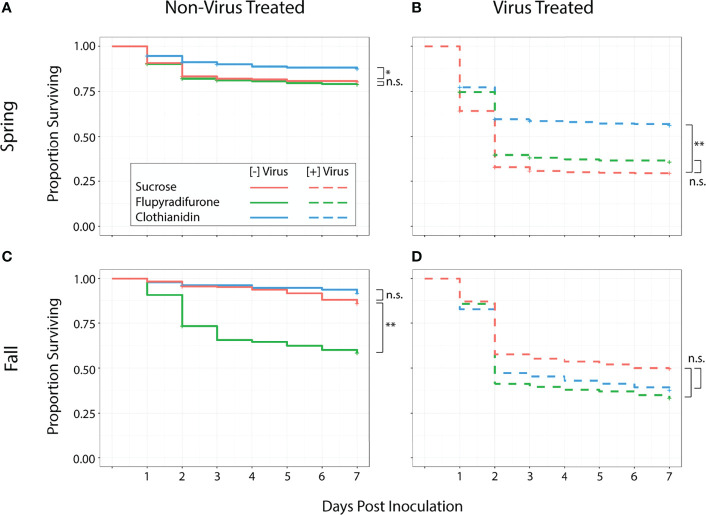
Survival of spring and fall honey bee workers exposed to pesticides, with or without virus. Total sample size N = 4530 (spring n = 2700, fall n = 2100). **(A)** In the spring, flupyradifurone consumption did not affect survival, while clothianidin consumption led to higher survival. **(B)** Springtime bees that consumed flupyradifurone+virus had similar survival to bees that consumed virus only, while bees that consumed clothianidin+virus had higher survival than those that consumed virus only. **(C)** In the fall, bees that consumed flupyradifurone had lower survival than those that consumed sucrose only, while bees that consumed clothianidin did not differ from the sucrose-only controls. **(D)** Consumption of either flupyradifurone or clothianidin did not affect virus-induced mortality in fall bees. Statistically significant differences are denoted with either * (0.01<P<0.05) or ** (P<0.01), while non-significant differences are denoted with n.s.

Among all treatments, individuals that consumed virus had lower survival than those that did not in both the spring (*z* = 13.15, *p <*0.001) and fall trials (*z* = 7.03, *p* < 0.001). Worker survival was affected by ingestion of pesticides and virus particles, but this effect was influenced by the season in which the trial occurred. Among springtime workers, ingestion of flupyradifurone did not affect survival. Workers that consumed flupyradifurone did not differ from those that consumed sucrose only (*z* = -0.32, *adj. p* = 0.75) (**Figure 1A**), and those that consumed flupyradifurone+virus did not differ from those that consumed virus only (*z* = 1.38, *adj. p* = 0.21) (**Figure 1B**). On the other hand, consumption of clothianidin showed a positive effect on survival. Springtime workers that consumed clothianidin had higher survival than those that consumed sucrose only (*z* = 2.57, *adj. p* = 0.017) (**Figure 1A**), and those that consumed clothianidin+virus also had higher survival than those that consumed virus only (*z* = 5.86, *adj. p* < 0.001) (**Figure 1B**).

In the fall, ingestion of flupyradifurone had a negative effect on worker survival. Those that ingested flupyradifurone had significantly lower survival than those that consumed only sucrose (*z* = -3.03, *adj. p* = 0.006) (**Figure 1C**), while those that consumed flupyradifurone+virus had a numerically lower but non-significant survival rate compared to those that consumed virus only (*z* = -1.98, *adj. p* = 0.08) (**Figure 1D**). Fall worker bees showed no adverse effects of consuming clothianidin, as clothianidin-fed workers did not differ from sucrose-fed workers (*z* = 1.1, *adj. p* = 0.27) (**Figure 1C**), and clothianidin+virus-fed workers did not differ from virus-fed workers (*z* = -1.16, *adj. p* = 0.27) (**Figure 1D**).

### Viral Titers

**Figure 2:**
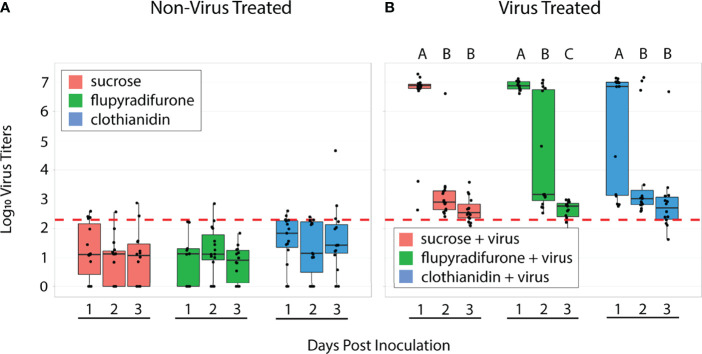
Viral titers of honey bee workers 1-, 2-, or 3-days post inoculation. **(A)** Bees not treated with IAPV showed low titers, with most individuals below the limit of detection. **(B)** Bees treated with virus showed varying levels of infection over the 3 days of sampling. In the virus-only treatment, the majority of individuals had high-level infection at day 1, but by day 2 and 3 nearly all individuals had low-level infection. In the flupyradifurone+virus treatment, all individuals were highly infected at day 1, while at day 2 there was a mix of individuals with high- and low-level infection. By day 3, flupyradifurone+virus treated bees all had low-level infection. In the clothianidin+virus treatment, most individuals had high-level infections at day 1, but by days 2 and 3 most individuals had low-level infection. Boxplots depict the median ± 1 s.e., with whiskers extending to 2 s.e. Black dots represent individual viral titers. Letters above bars denote pairwise significance comparisons within each treatment group. The red dashed line depicts the theoretical limit of detection of the qPCR instrument. N = 162 samples total, with n = 39-45 per treatment group.

We measured viral titers *via* RT-qPCR using samples collected from each cage of our spring experiments over 3 days (n = 10-15 samples per treatment per day). As expected, treatment groups that did not ingest IAPV showed minimal viral titers, with most samples near or below the limit of detection (**Figure 2A**). This confirms that bees used in this experiment did not generally have background infections of IAPV. Among virus-treated workers, viral titers over the 3 days of sampling differed between treatment groups (**Table 1**). At 1 day post inoculation, most individuals in all 3 treatment groups showed high-level IAPV infection (flupyradifurone+virus vs virus-only *adj. p* = 0.369; clothianidin+virus vs virus-only *adj. p* = 0.111). However, at 2 days post inoculation, the flupyradifurone+virus treatment group had a greater proportion of individuals still maintaining high-level IAPV infection than the virus-only treatment, but the clothianidin+virus treatment did not (flupyradifurone+virus vs virus-only *adj. p* < 0.001; clothianidin+virus vs virus-only *adj. p* = 0.111). By 3 days post inoculation, most individuals in all treatment groups showed low-level IAPV infection (flupyradifurone+virus vs virus-only *adj. p* = 1.0; clothianidin+virus vs virus-only *adj. p* = 0.359) (**Figure 2B**).

**Table 1:** Pairwise comparisons of viral titers between treatments and days after viral inoculation, using binomial exact tests and Benjamini-Hochberg corrections for multiple comparisons.

Pairwise Statistical Comparisons of Viral Titers			
Between Treatments; Within Days	P-value	Within Treatments; Between Days	P-value
*Day 1*		*Virus only*	
Flupyradifurone+Virus vs Virus only	0.369	Day 1 vs Day 2	**<0.001**
Clothianidin+Virus vs Virus only	0.111	Day 2 vs Day 3	0.619
*Day 2*		Day 1 vs Day3	**<0.001**
Flupyradifurone+Virus vs Virus only	**<0.001**	*Flupyradifurone+Virus*	
Clothianidin+Virus vs Virus only	0.111	Day 1 vs Day 2	**<0.001**
*Day 3*		Day 2 vs Day 3	**0.005**
Flupyradifurone+Virus vs Virus only	1	Day 1 vs Day3	**<0.001**
Clothianidin+Virus vs Virus only	0.359	*Clothianidin+Virus*	
		Day 1 vs Day 2	**0.001**
		Day 2 vs Day 3	0.373
		Day 1 vs Day3	**<0.001**

Bold numbers denotes statistically significant differences between groups.

## Discussion

Evaluating the efficacy and safety of novel pest suppression chemistries requires careful consideration of the context in which the pesticides are administered. Flupyradifurone and the neonicotinoids that preceded it both act systemically and target acetylcholine receptors, but despite these similarities, honey bee workers respond differently to flupyradifurone and clothianidin. Our results indicate that there are substantial seasonal effects of pesticide-induced mortality. In the spring, bees exposed to flupyradifurone survived as well as control-treated bees, which is consistent with industry studies demonstrating low toxicity of this new chemistry (27). However, bees exposed to the same dose of flupyradifurone in the fall displayed significantly lower survival than control-treated bees. This corroborates studies from other groups which found seasonal effects of flupyradifurone toxicity on honey bees (33). While there are many biotic and abiotic factors that differ in the spring and fall, perhaps the most relevant is the availability of different flowering plants for forage. Pollen, the main source of dietary protein and lipids, has different nutrient profiles across different seasons, and this can affect worker development and ability to combat infections (57), as well as their overwintering success (58). Likewise, bees display seasonal variation in their sensitivity to pesticides (40, 59), and diet has been directly linked to pesticide tolerance (60). In fact, consuming pollen increases expression of detoxification enzymes and can increase bee survival when exposed to pesticides (61, 62). Our results here provide further evidence that honey bee sensitivity to some pesticides may be seasonally dependent and illustrate that restricting toxicity studies to a single season can create gaps in our knowledge. This may be particularly important for assessing the risk of pesticides used across different crops in different regions, where applications and thus bee exposure may differ. Because the effects on bees may be seasonally variable, a harmless exposure in one context may have larger ramifications in another. Further, because systemic pesticides can remain in plant tissue and soils for months (63, 64), plant materials foraged and stored by a colony (including nectar, pollen, and propolis) could result in low-level exposure throughout the year (65).

Another important finding was that clothianidin exposure appears to confer some benefit to bees in the springtime, at least at the dosage used here. Bees given clothianidin had ~7% higher survival than bees given the sucrose-only control, while bees given clothianidin+virus had ~26% higher survival than bees given the virus-only treatment, suggesting that clothianidin improves response to IAPV infection in bees during this time. Like other pesticides studied, clothianidin exposure shows a mix of both negative and neutral effects, and sometimes even positive outcomes for honey bees. On the one hand, clothianidin can impair worker memory (66), alter foraging and communication (67), diminish immune responses *via* lower hemocyte counts and decreased antimicrobial activity (68), and increase viral replication of DWV (23). On the other hand, clothianidin has shown to have no adverse effects on mortality, longevity, or overwintering success of honey bees (69). The higher survival of clothianidin-treated bees that we observed is likely due to hormesis, where consumption of small amounts of xenobiotics such as pesticides can activate detoxification and immunological pathways and increase bees’ resistance to chemical and biological stressors (47, 70–72). This corroborates other studies showing hormetic effects of clothianidin, including increased enzymatic activity of cytochrome P450s in honey bees (73), increased colony growth in bumble bees (74), and increased longevity in mason bees (75). This illustrates that sublethal doses of pesticides can elicit varied responses in off-target organisms. While much focus is aimed at understanding acute toxicity and deleterious effects on pollinators, it is important to highlight instances where hormetic effects of pesticide exposure may actually aid important physiological processes like detoxification and immunity. This provides a more complete picture of pesticide effects when weighing the costs and benefits of applying certain pest suppression strategies.

This study also shows that pesticides can have varied, often subtle, effects on honey bees. The significance of these subtle effects is hard to discern, but it illustrates that there are more factors to consider than just acute toxicity and LD50 measurements. Here, we observed that exposure to flupyradifurone may impair bees’ ability to clear a viral infection. When inoculated with IAPV, bees sampled at 24 hrs show high viral titers, but those sampled at 48 and 72 hrs post-inoculation have greatly reduced viral titers. However, when viral inoculation is paired with exposure to flupyradifurone, a greater proportion of bees sampled at 48 hours post inoculation maintain high viral titers. This is somewhat inconsistent with other recent studies examining flupyradifurone’s effect on 3 other honey bee viruses (76). In that study, flupyradifurone exposure did not affect titers of BQCV or DWV-A and -B at 7- and 14-days post inoculation. However, IAPV may have different host-pathogen interactions, as our data show bees not exposed to flupyradifurone are mostly free of infection within 2 days. Even though flupyradifurone appears to delay virus clearing, this did not lead to an increase in mortality among the relevant treatment groups. Viral titers were unaffected by clothianidin, as both clothianidin-treated and control-treated groups had similar proportions of individuals with high-level infections over the 3 days of sampling. This is somewhat contradictory to previous studies, in which a comparable dose of clothianidin led to increased replication of DWV (23). Thus, pesticide effects on antiviral immunity may be both pathogen-specific and pesticide-specific and could have ramifications on transmission dynamics. It should be noted there are challenges with measuring viral clearance. It is possible that lower titers observed in individuals sampled at days 2 and 3 are due to some degree of survivor bias, but given the prevalence of highly infected individuals sampled at random on day 1 this effect is likely minimal. An alternative strategy would be to re-measure the same individuals each day by extracting hemolymph samples, but the stress of repeatedly subjecting individuals to this type of treatment would likely affect their viral titers and overall health.

Taken together, our results highlight that pesticide effects on honey bees and other off-target organisms can be subtle and context-dependent. As the next generation of “pollinator-friendly” pesticides come to market, increased scientific scrutiny of these novel chemistries will inevitably reveal hitherto unknown sublethal effects. In the big picture, it will be important to assess how impactful these off-target effects are, and what alternative options are available for pest management. If approval of these novel chemistries is revoked or increased regulatory oversight prevents their registration altogether, growers may turn to older products, many of which are also demonstrated as unsafe for bees or are poorly studied. In the meantime, it is critical that we continue to investigate the broad array of sublethal effects caused by pesticides so that we can better inform policy and decision-making.

## Data Availability Statement

The original contributions presented in the study are included in the article/supplementary material. Further inquiries can be directed to the corresponding author.

## Author Contributions

GH: conceptualization, formal analysis, investigation, methodology, writing (original draft, review, editing). VP: investigation, methodology, formal analysis. AD: project administration, funding acquisition, conceptualization, methodology, writing (review and editing). All authors contributed to the articled and approved the submitted version.

## Funding

This work was funded by the US Department of Agriculture grant 2019-67013-29300.

## Conflict of Interest

The authors declare that the research was conducted in the absence of any commercial or financial relationships that could be construed as a potential conflict of interest.

## Publisher’s Note

All claims expressed in this article are solely those of the authors and do not necessarily represent those of their affiliated organizations, or those of the publisher, the editors and the reviewers. Any product that may be evaluated in this article, or claim that may be made by its manufacturer, is not guaranteed or endorsed by the publisher.
